# Clinicopathological Differences in HER2 Immunohistochemical Expression Between Low-Grade Endometrial Cancer with p53-Abnormal Expression and High-Grade Endometrial Cancer

**DOI:** 10.3390/cancers18162638

**Published:** 2026-08-15

**Authors:** Kazuhisa Hachisuga, Miya Nakashima, Yoshihiro Katayama, Yusuke Inomata, Hiroshi Tomonobe, Shoji Maenohara, Keisuke Kodama, Hiroshi Yagi, Ichiro Onoyama, Kazuo Asanoma, Yoshinao Oda, Hideaki Yahata

**Affiliations:** 1 Department of Gynecology and Obstetrics, Graduate School of Medical Sciences, Kyushu University, Fukuoka 812-8582, Japaninomata.yusuke.218@m.kyushu-u.ac.jp (Y.I.);; 2 Department of Anatomic Pathology, Graduate School of Medical Sciences, Kyushu University, Fukuoka 812-8582, Japan

**Keywords:** endometrial carcinoma, clinicopathological feature, p53-abnormal expression, HER2 expression

## Abstract

**Background/Objectives:** Under the current molecular classification of endometrial cancer, the p53-abnormal group is defined as having the worst prognosis, but the clinicopathological position of low-grade endometrial cancer with p53-abnormal expression relative to high-grade endometrial cancer remains unclear. We previously showed that low-grade endometrial cancer with p53-abnormal expression more closely resembles low-grade endometrial cancer with p53 wild-type expression than high-grade endometrial cancer and that the *ERBB2* gene, encoding Human Epidermal Growth Factor Receptor 2 (HER2) protein, is more highly expressed in high-grade endometrial cancer. This study compared HER2 immunohistochemical expression (IHC) among low-grade endometrial cancer with wild-type p53 expression (EC^lo^p53^wt^), low-grade endometrial cancer with p53-abnormal expression (EC^lo^p53^ab^), and high-grade endometrial cancer (EC^hi^), in order to characterize EC^lo^p53^ab^. **Methods:** We retrospectively analyzed 70 endometrial cancer cases treated at Kyushu University Hospital in 1992–2022. Tumors were classified as EC^lo^p53^wt^, EC^lo^p53^ab^, and EC^hi^ based on histopathology and p53 immunohistochemistry, with EC^hi^ corresponding to conventional uterine serous carcinoma. HER2 expression was evaluated immunohistochemically using a standardized scoring system (0, 1+, 2+, 3+), and its distribution was compared across the three groups, together with clinicopathological parameters and patient outcomes. **Results:** EC^hi^ showed the highest frequency and intensity of HER2 expression, with a substantially larger proportion of HER2 IHC 3+ cases than EC^lo^p53^wt^ and EC^lo^p53^ab^ (*p* < 0.0001 and *p* = 0.0146, respectively). Meanwhile, there was no significant association between EC^lo^p53^wt^ and EC^lo^p53^ab^ (*p* = 0.3794). In addition, HER2 IHC 3+ had significant associations with older age (≥60 years) and substantial lymphovascular space invasion (*p* = 0.0003 and 0.0174, respectively). **Conclusions:** EC^lo^p53^ab^ exhibits HER2 profiles and clinical behavior more similar to EC^lo^p53^wt^, supporting the more nuanced application of molecular classification and HER2-targeted therapy in endometrial cancer.

## Simple Summary

This study compared HER2 immunohistochemical expression (IHC) among three endometrial cancer subgroups: low-grade endometrial cancer with wild-type p53 expression (EC^lo^p53^wt^), low-grade endometrial cancer with p53-abnormal expression (EC^lo^p53^ab^), and high-grade endometrial cancer (EC^hi^). HER2 IHC 3+ was significantly more frequent in the EC^hi^ group than in the EC^lo^p53^ab^ group, while no significant difference was observed between the EC^lo^p53^wt^ and EC^lo^p53^ab^ groups. HER2 IHC 3+ was associated with older age and substantial lymphovascular space invasion and identified patients with significantly shorter progression-free and overall survival. These findings suggest that, despite sharing p53-abnormal expression, EC^hi^ represents a biologically aggressive subset distinct from EC^lo^p53^ab^, in which HER2-targeted therapies, including trastuzumab deruxtecan, may be particularly relevant. Careful HER2 scoring may therefore help refine risk stratification and guide biomarker-driven treatment strategies in endometrial cancer.

## 1. Introduction

In 2013, The Cancer Genome Atlas (TCGA) Research Network reported a comprehensive genomic and transcriptomic analysis of endometrial carcinoma and identified four molecular subgroups: *POLE*-ultramutated, microsatellite instability-high, copy-number-low, and copy-number-high tumors. Among these, copy-number-high tumors are characterized by frequent *TP53* mutations and have the worst prognosis [1]. Building on these findings, Kommoss et al. developed the Proactive Molecular Risk Classifier for Endometrial Cancer [2], and this molecular classification has since been incorporated into the ESGO/ESTRO/ESP guidelines 2021 and the International Federation of Gynecology and Obstetrics (FIGO) 2023 staging system [3,4].

Although molecular classification has improved risk stratification in endometrial carcinoma, various issues remain unresolved in its application in routine clinical practice. For example, *POLE* mutation analysis is required for molecular classification, but it is costly and may be difficult to apply in some clinical settings. In addition, the clinicopathological significance of p53-abnormal expression remains incompletely understood, particularly in low-grade endometrial carcinoma. We previously demonstrated that low-grade endometrial carcinoma with p53-abnormal expression was more similar to low-grade endometrial carcinoma with wild-type p53 expression than to high-grade endometrial carcinoma in terms of immunohistochemical and RNA-sequencing results [5]. Similarly, Vrede et al. reported that low-grade endometrial cancer had an excellent prognosis independent of molecular subgroup [6]. However, Jamieson et al. demonstrated that p53-abnormal, low-grade endometrial cancers are at substantial risk of recurrence [7]. These findings suggest that the clinical significance of molecular classification, especially within the p53-abnormal group in low-grade endometrial carcinoma, remains to be fully clarified.

Human Epidermal Growth Factor Receptor 2 (HER2)-targeted antibody–drug conjugates (ADCs) have emerged as promising therapies for endometrial carcinoma. In the DESTINY-PanTumor02 trial, trastuzumab deruxtecan (T-DXd) achieved an overall response rate (ORR) of 57.5% in the endometrial cancer cohort [8]. Notably, in patients with confirmed HER2 immunohistochemical expression (IHC) 3+, the ORR was 84.6%. In addition, in our previous study, the *ERBB2* gene, which encodes the HER2 protein, was more highly expressed in high-grade endometrial carcinoma than in low-grade endometrial carcinoma with p53-abnormal expression [5].

Against this background, we evaluated HER2 IHC in low-grade endometrial carcinoma with p53-abnormal expression, high-grade endometrial carcinoma, and low-grade endometrial carcinoma with wild-type p53 expression. The main aim here was to clarify the clinicopathological position of low-grade endometrial carcinoma with p53-abnormal expression relative to high-grade endometrial carcinoma, as well as to explore the significance of HER2 expression in endometrial carcinoma.

In particular, we focused on low-grade p53-abnormal tumors that were MMR-proficient and lacked pathogenic *POLE* hotspot mutations, in order to clarify whether HER2 expression identifies a biologically aggressive subset within this group or is predominantly restricted to p53-abnormal high-grade disease.

## 2. Methods

### 2.1. Case Selection and Clinicopathological Characteristics

All samples used in this study were collected at the Department of Gynecology and Obstetrics, Kyushu University, from 1992 to 2022. In this study, we defined low-grade endometrial cancer with wild-type p53 expression, low-grade endometrial cancer with p53-abnormal expression, and high-grade endometrial cancer as the EC^lo^p53^wt^ group, EC^lo^p53^ab^ group, and EC^hi^ group, respectively. The two EC^lo^ groups were histologically confirmed to involve endometrioid carcinoma grades 1–2 (G1–2) and retained diffuse ER expression. In terms of molecular classification, all EC^lo^p53^ab^ tumors showed retained nuclear expression of both PMS2 and MSH6 on immunohistochemistry, indicating MMR proficiency, and all 13 EC^lo^p53^ab^ tumors were tested for *POLE* sequencing. *POLE* sequencing was focused on the exonuclease domain hotspot mutations P286R and V411L, which are the most prevalent pathogenic *POLE* alterations in endometrial cancer [9] and correspond to the hotspot regions evaluated in our previous study [10]. According to the FIGO 2023 molecular classification, MMR status was assessed by immunohistochemistry for PMS2 and MSH6, and retained nuclear expression of both proteins was used as evidence of MMR proficiency [4]. Thus, the EC^lo^p53^ab^ group in this study corresponds to the p53-abnormal, MMR-proficient, non-*POLE* molecular category. Meanwhile, the EC^hi^ group was characterized by severe nuclear atypia, papillary structure, p53-abnormal expression, and decreased ER expression, reflecting conventional uterine serous carcinoma.

All EC^hi^ tumors included in this study showed a p53-abnormal immunostaining pattern on immunohistochemistry. In molecular classification, uterine corpus serous carcinoma was reported to be mostly classified into the p53-abnormal group [1]. Histological type and FIGO grade were assigned by board-certified gynecologic pathologists at our institution according to contemporaneous WHO/FIGO criteria.

We reviewed 70 Japanese patients with histologically and immunohistochemically confirmed endometrial cancer (EC^lo^p53^wt^ group: *n* = 32, EC^lo^p53^ab^ group: *n* = 13, and EC^hi^ group: *n* = 25). The medical records and hematoxylin and eosin (HE)-stained slides were re-reviewed to obtain clinical and pathological data regarding age, FIGO 2009 stage, metastasis, lymphovascular space invasion (LVSI), myometrial invasion depth, uterine cervical invasion, peritoneal cytology, therapeutic methods, recurrence, progression-free survival (PFS), and overall survival (OS).

With regard to age, ≥60 years is here defined as elderly, while lymph node metastasis is subclassified based on the amount of tumor tissue as follows: isolated tumor cells (ITCs) (≥0.2 mm and ≤200 cells), or micro- (0.2–2 mm and/or >200 cells) or macrometastasis (>2 mm) [11]. Meanwhile, LVSI is subclassified as none, focal, or substantial. Substantial LVSI is defined as the involvement of five or more vessels, while focal LVSI is defined as that of fewer than five vessels [12]. Lymphadenectomy, when performed, was defined as pelvic lymphadenectomy with or without para-aortic lymphadenectomy.

All procedures were performed in accordance with relevant guidelines and regulations. Informed consent was waived, and an opt-out approach was used. The institutional review board of Kyushu University approved this study (project number: 25196).

### 2.2. Immunohistochemical Staining

#### 2.2.1. Antibodies

The following primary antibodies were used: p53 (mouse monoclonal, clone DO-7, dilution 1:100; Novocastra, Newcastle, UK), ER (mouse monoclonal, clone 6F11, dilution 1:50; Novocastra), HER2 (polyclonal, clone A0485, dilution 1:200; Dako, Glostrup, Denmark), PMS2 (mouse monoclonal, clone A16-4, dilution 1:200; BD Biosciences, Heidelberg, Germany), and MSH6 (rabbit monoclonal, clone EP49, dilution 1:200; Dako).

#### 2.2.2. Procedure

Immunostaining was performed on 4-µm sections cut from silane-coated slides using the universal immunoperoxidase polymer method (Envision kit; Dako) in accordance with the manufacturer’s instructions. After antigen retrieval by microwave heating and pressure cooking, the sections were allowed to cool, then deparaffinized, rehydrated, and treated to block endogenous peroxidase activity. The primary antibodies were applied at room temperature for 90 min, followed by incubation with the secondary antibody. The sections were then developed with 3,3′-diaminobenzidine, counterstained with hematoxylin, mounted, and examined. Positive and negative controls were included in each run.

#### 2.2.3. Immunohistochemical Scoring

p53-abnormal expression was defined when more than 80% of tumor cell nuclei showed strong staining, when nuclear staining was completely absent in tumor cells with intact staining in internal controls [13], or when a distinct cytoplasmic p53 staining pattern was present [14].

HER2 immunohistochemistry was evaluated on surgically resected specimens and scored according to the gastric cancer-specific criteria proposed by Bartley et al. [15]. Tumors were classified as IHC 0 (no reactivity or membranous reactivity in less than 10% of tumor cells), 1+ (faint or barely perceptible membranous reactivity in at least 10% of tumor cells, with reactivity observed in only part of the membrane), 2+ (weak-to-moderate complete, basolateral, or lateral membranous reactivity in at least 10% of tumor cells), or 3+ (strong complete, basolateral, or lateral membranous reactivity in at least 10% of tumor cells). In line with recent pan-tumor trials, cases with IHC 2+ or 3+ were considered HER2-positive. The expression of PMS2 or MSH6 was considered “lost” when nuclear staining was completely absent in tumor cells, whereas the surrounding normal cells showed consistently preserved nuclear staining. Representative images of PMS2 and MSH6 expression are provided in Supplementary Figure S1.

The assessment of IHC was carried out independently by a pathologist and a physician specializing in gynecological oncology, both of whom were blinded to the clinicopathological details of the patients. No automated image-analysis software was used. For each case, at least several non-overlapping high-power fields were reviewed, and the percentage of positive tumor cells and staining intensity were estimated visually according to the predefined criteria. In cases of discrepant scores, a consensus was reached by joint review using a multi-headed microscope (Evident, Tokyo, Japan).

### 2.3. Statistical Analysis

We used the statistical software program JMP^®^ 18 (SAS Institute, Cary, NC, USA) for all statistical analyses. Pearson’s chi-squared test and Fisher’s exact test were used to analyze the data. Kaplan–Meier analysis and the log-rank test were used for survival analysis. In all analyses, a *p*-value of <0.05 was considered statistically significant.

## 3. Results

### 3.1. Clinicopathological Characteristics

In this study, we reviewed 70 cases (EC^lo^p53^wt^ group: 32 cases, EC^lo^p53^ab^ group: 13 cases, and EC^hi^ group: 25 cases), the clinicopathological features of which are summarized in Table 1. After carefully reviewing the clinical data of these cases, we reclassified all of them based on the FIGO 2023 staging. Upon this reclassification, only one case in the EC^lo^p53^wt^ group was reclassified from stage IIIA to stage IA3 and was categorized as being at an early stage, not an advanced stage. Regarding LVSI, 15 cases in this study were classified as substantial (EC^lo^p53^wt^ group: 8 cases, EC^lo^p53^ab^ group: 1 case, and EC^hi^ group: 6 cases). Recurrence was observed in 17 cases (EC^lo^p53^wt^ group: 2 cases, EC^lo^p53^ab^ group: 3 cases, and EC^hi^ group: 12 cases). As for treatment, all patients underwent total hysterectomy and 63 (EC^lo^p53^wt^ group: 29 cases, EC^lo^p53^ab^ group: 13 cases, and EC^hi^ group: 21 cases) also underwent lymphadenectomy in this study. By definition, EC^lo^p53^wt^ tumors showed a p53-wild-type staining pattern and EC^lo^p53^ab^ tumors showed a p53-abnormal pattern. All EC^hi^ tumors were also confirmed to be p53-abnormal by immunohistochemistry.

### 3.2. Immunohistochemical Analysis

**Figure 1:**
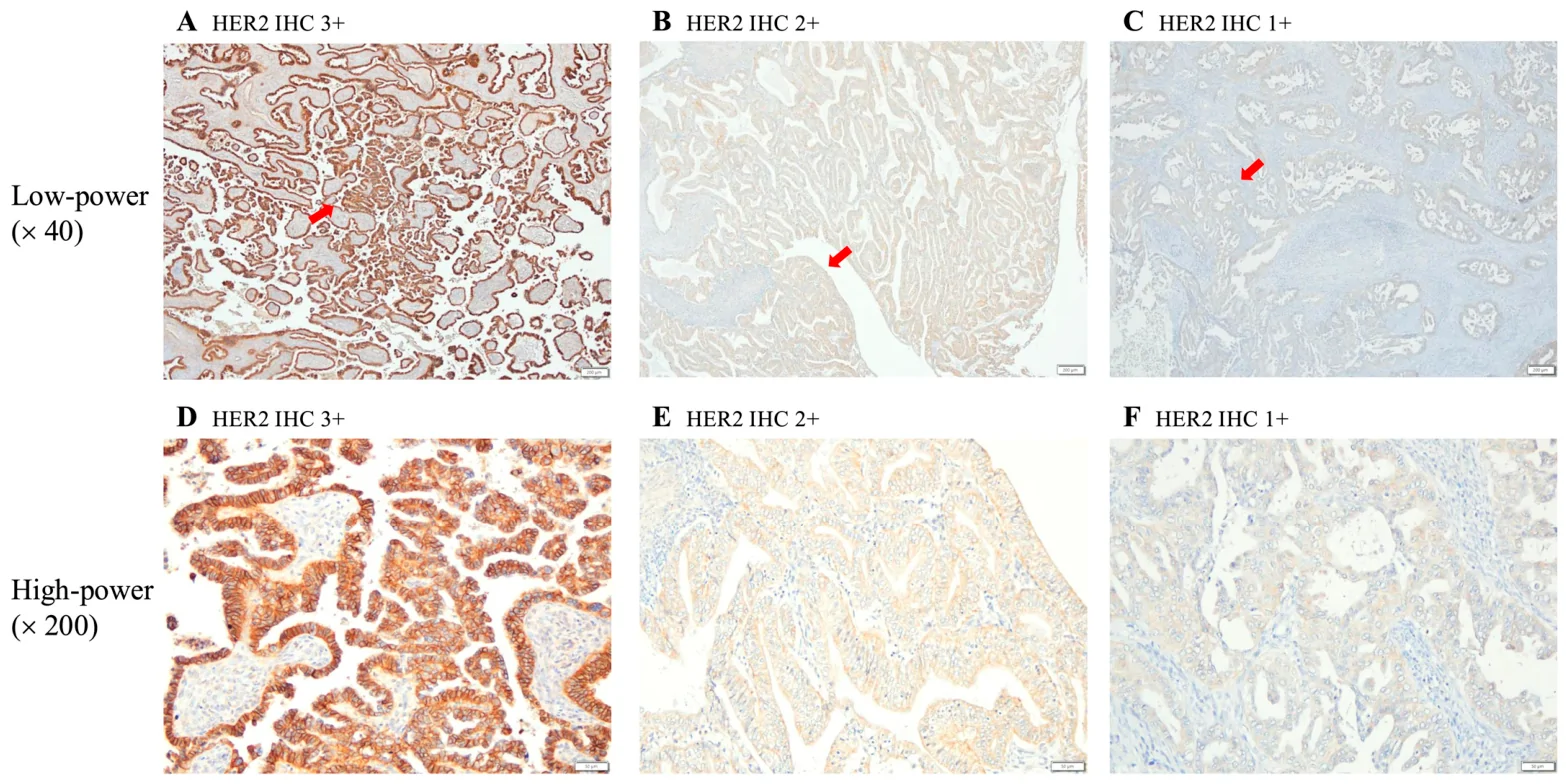
Representative HER2 immunohistochemical staining patterns according to HER2 score in endometrial cancer. (**A**) Low-power view of a tumor with HER2 IHC 3+. The arrow indicates the area shown at higher magnification in (**D**). (**B**) Low-power view of a tumor with HER2 IHC 2+. The arrow indicates the area shown at higher magnification in (**E**). (**C**) Low-power view of a tumor with HER2 IHC 1+. The arrow indicates the area shown at higher magnification in (**F**). (**D**) High-power view of the area indicated by the arrow in (**A**), showing strong complete, basolateral, or lateral membranous staining. (**E**) High-power view of the area indicated by the arrow in (**B**), showing weak-to-moderate complete, basolateral, or lateral membranous staining. (**F**) High-power view of the area indicated by the arrow in (**C**), showing faint or barely perceptible incomplete membranous staining. Scale bars: 200 μm in (**A**–**C**) and 50 μm in (**D**–**F**).

**Figure 2:**
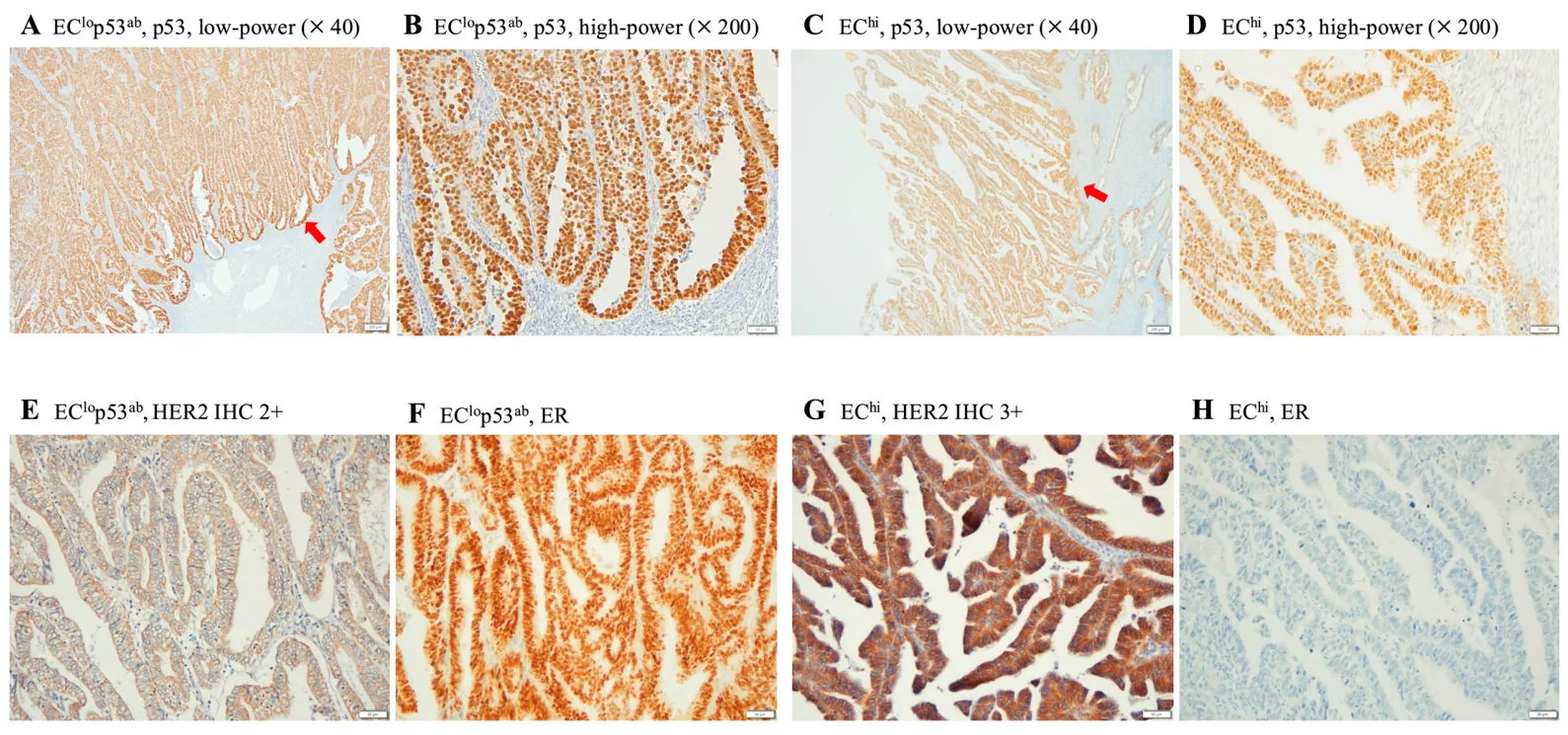
Representative immunohistochemical staining patterns in low-grade endometrial cancer with p53-abnormal expression (EC^lo^p53^ab^) and high-grade endometrial cancer (EC^hi^). (**A**,**B**) p53 immunostaining in an EC^lo^p53^ab^ tumor. (**A**) Low-power view and (**B**) high-power view of the area indicated by the arrow in (**A**), showing diffuse strong nuclear p53 staining consistent with a p53-abnormal pattern. (**C**,**D**) p53 immunostaining in an EC^hi^ tumor. (**C**) Low-power view and (**D**) high-power view of the area indicated by the arrow in (**C**), also showing diffuse strong nuclear p53 staining consistent with a p53-abnormal pattern. (**E**) HER2 immunostaining in the EC^lo^p53^ab^ tumor, showing an IHC score of 2+. (**F**) ER immunostaining in the EC^lo^p53^ab^ tumor, showing preserved diffuse nuclear ER expression. (**G**) HER2 immunostaining in the EC^hi^ tumor, showing strong complete, basolateral, or lateral membranous staining with an IHC score of 3+. (**H**) ER immunostaining in the EC^hi^ tumor, showing reduced ER expression. Scale bars: 200 μm in (**A**,**C**), and 50 μm in (**B**) and (**D**–**H**).

Representative immunohistochemical images of HER2 are shown in Figure 1. In addition, representative immunohistochemical images of HER2, p53, and ER for EC^lo^p53 ^ab^ and EC^hi^ groups are shown in Figure 2. Although the apparent intensity of p53 staining varied to some extent among individual tumors, both the EC^lo^p53^ab^ and EC^hi^ groups consistently showed a p53-abnormal immunostaining pattern (either diffuse strong overexpression or complete absence with intact internal controls), rather than a wild-type pattern. The distributions of HER2 IHC are shown in Table 2, Table 3 and Table 4. In addition, these distributions are depicted graphically in Supplementary Figure S2, which highlights the higher proportion of HER2 IHC 3+ tumors in the EC^hi^ group compared with the EC^lo^p53^wt^ and EC^lo^p53^ab^ groups. Regarding HER2 IHC 3+, there were 6 such cases (18.8%) in the EC^lo^p53^wt^ group, 4 (30.8%) in the EC^lo^p53^ab^ group, and 18 (72.0%) in the EC^hi^ group. As for cases with HER2-positive expression, there were 20 such cases (62.5%) in the EC^lo^p53^wt^ group, 12 (92.3%) in the EC^lo^p53^ab^ group, and 24 (96.0%) in the EC^hi^ group. Significantly fewer cases of the EC^lo^p53^ab^ group showed HER2 IHC 3+ than in the EC^hi^ group (*p* = 0.0146). However, there was no significant difference in HER2 IHC 3+ between the EC^lo^p53^wt^ group and the EC^lo^p53^ab^ group (*p* = 0.3794). On the other hand, HER2 IHC 3+ was significantly associated with both older age (≥60 years; *p* = 0.0003) and substantial LVSI (*p* = 0.0174) (Table S1).

### 3.3. Survival Analysis

**Figure 3:**
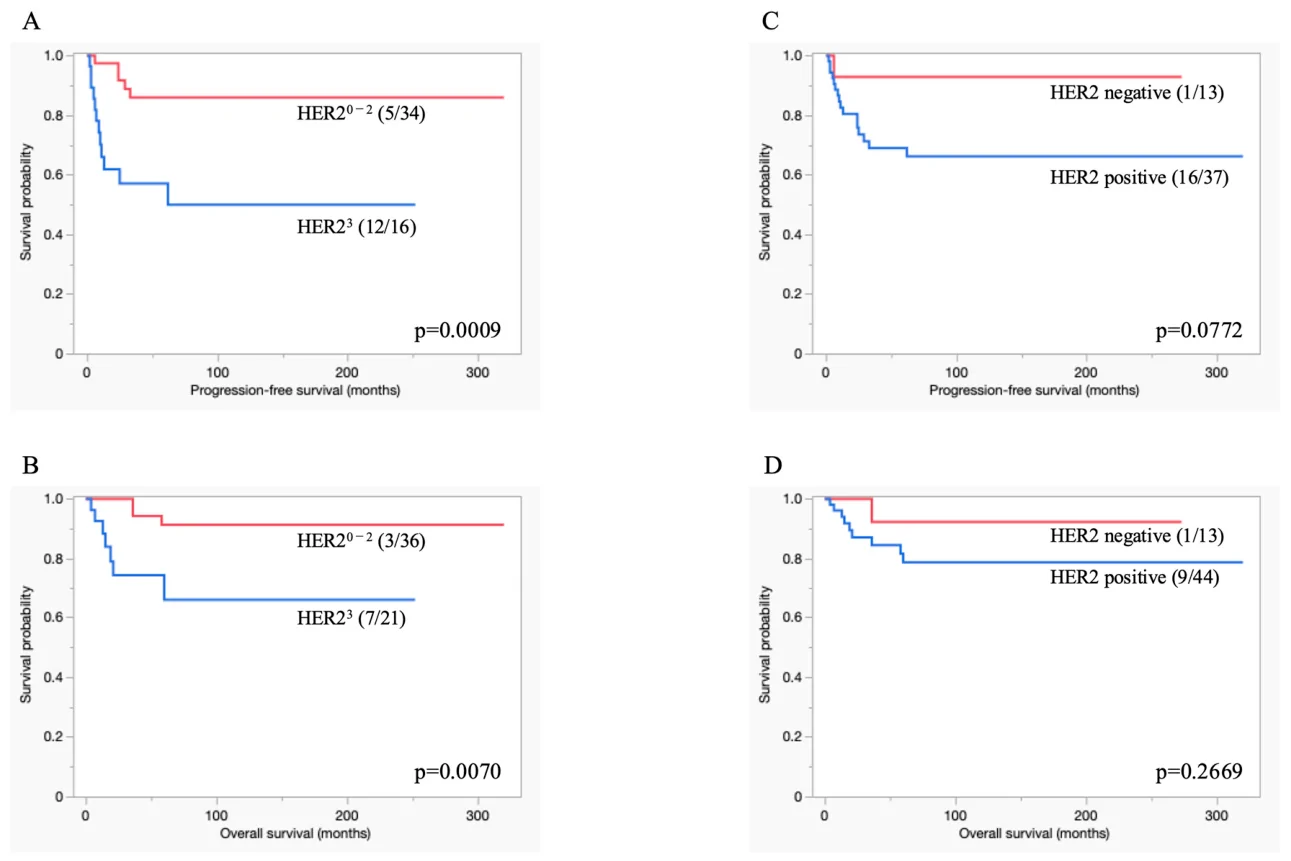
Kaplan–Meier curves for progression-free survival and overall survival according to HER2 immunohistochemical status. (**A**,**B**) Progression-free survival and overall survival according to HER2 IHC score, comparing HER2 IHC 3 and HER2 IHC 0–2 cases. (**C**,**D**) Progression-free survival and overall survival according to HER2 status, comparing HER2-positive and HER2-negative cases. Numbers in parentheses indicate events/number at risk (recurrence for progression-free survival and death for overall survival). *p* values were calculated using the log-rank test.

Figure 3 shows the results of Kaplan–Meier analyses for PFS and OS. The PFS and OS periods were significantly shorter in the group with HER2 IHC 3+ than in the rest of the patients (*p* = 0.0009 and *p* = 0.0070, respectively). However, there were no significant associations between HER2-positive IHC and PFS or OS (*p* = 0.0772 and *p* = 0.2669, respectively).

## 4. Discussion

In this study, we evaluated HER2 IHC in endometrial cancer. Significantly fewer cases of the EC^lo^p53^ab^ group showed HER2 IHC 3+ than in the EC^hi^ group. Meanwhile, there was no significant difference in the rate of HER2 IHC 3+ between the EC^lo^p53^wt^ group and the EC^lo^p53^ab^ group. In survival analyses, the PFS and OS periods were significantly shorter in the group with HER2 IHC 3+ than in the rest of the patients. However, HER2-positive IHC showed no significant association with PFS or OS. These findings indicate that, even within the p53-abnormal group, HER2 IHC 3+ characterizes a biologically aggressive subset predominantly represented by the EC^hi^ group and suggest that the EC^lo^p53^ab^ group is closer to the EC^lo^p53^wt^ group than to the EC^hi^ group clinicopathologically.

The molecular classification for endometrial cancer has been incorporated into FIGO 2023 staging and ESGO guidelines 2021 [3,4]. In this molecular classification, the p53-abnormal group has the worst prognosis. However, the clinical significance of p53-abnormal expression is still unclear. In fact, Vrede et al. suggested that a routine molecular classification for low-grade endometrial cancer may not be needed [6]. In our previous study, we demonstrated that the EC^lo^p53^ab^ group was more similar biologically to the EC^lo^p53^wt^ group than to the EC^hi^ group [5]. In terms of other molecular characteristics, RNA sequencing analysis showed that the *ERBB2* gene was significantly more highly expressed in the EC^hi^ group than in the EC^lo^p53^ab^ group, which is consistent with the results of our study. In addition, the EC^hi^ group showed the worst prognosis in our previous study. In view of such unfavorable outcomes, personalized treatment strategies for the p53-abnormal group, traditionally represented by uterine serous carcinoma, have attracted increasing attention. For example, tailored treatment was applied for the p53-abnormal group in the RAINBO trial, with the results attracting considerable attention [16], and such individualized strategies are now being increasingly investigated.

In the present study, the EC^hi^ group showed significantly more frequent HER2 IHC 3+ than the others. This is concordant with prior evidence showing that HER2 overexpression is enriched in biologically aggressive tumors, including uterine serous carcinoma [17,18]. HER2, a targetable transmembrane glycoprotein receptor of the Epidermal Growth Factor Receptor (EGFR) family, plays an important role in cell proliferation, survival, and differentiation [19]. Aberrant HER2 signaling has been implicated in various cancers, particularly gastric and breast cancers, in which HER2 overexpression or amplification is associated with aggressive tumor behavior and poor prognosis [19,20]. It has also been reported that HER2-activating mutations contribute to accelerated tumorigenesis and metastasis [21]. Specifically, HER2 overexpression in cancer cells results in an excess of HER2 protein in the membranes of these cells [19]. This overexpression can lead to uncontrolled cell growth and is associated with more aggressive forms of cancer. It was also previously reported that HER2 IHC is associated with higher tumor grade and unfavorable prognosis in various cancers [19,20,22]. In addition, in endometrial cancer in particular, it was reported that there was an association between uterine serous carcinoma and HER2 immunohistochemical overexpression [17]. Serous carcinoma, which is represented by the EC^hi^ group in this study, has traditionally been regarded as a poor-prognosis subtype of endometrial carcinoma, for which novel therapeutic strategies are still needed. Against this background, the findings of the current study suggest that an ADC targeting the HER2 protein could be a promising treatment for patients in the EC^hi^ group. Previous studies have reported that HER2 amplification and overexpression frequently co-occur with *TP53* mutations in high-grade endometrial carcinomas, including serous and high-grade endometrioid tumors, and that this subgroup may benefit from HER2-targeted therapy [23]. Our findings are in line with these observations in that strong HER2 IHC 3+ was largely confined to the EC^hi^ tumors. However, by directly comparing EC^lo^p53^ab^ with EC^hi^ and EC^lo^p53^wt^, our study further demonstrates that EC^lo^p53^ab^ tumors rarely show HER2 IHC 3+ and are clinicopathologically closer to EC^lo^p53^wt^ than to EC^hi^. These results suggest that HER2 IHC 3+ identifies a particularly aggressive subset within the p53-abnormal group that is largely restricted to high-grade disease.

Another important implication of the present study relates to the methodology and interpretation of HER2 testing in endometrial carcinoma. Although HER2 has become an established therapeutic biomarker in uterine serous carcinoma, the approach for assessing HER2 in endometrial cancer remains less standardized than in breast and gastric cancers, and interlaboratory variation in testing and reporting procedures has been recognized [24,25,26]. In particular, uterine serous carcinoma frequently exhibits basolateral or lateral membranous staining, intratumoral heterogeneity, and occasional discordance between immunohistochemistry and in situ hybridization, all of which can complicate t the clinically meaningful identification of HER2-positive cases [17,27,28]. In the present study, HER2 immunohistochemistry was scored using gastric cancer-specific criteria proposed by Bartley et al. [15] because these criteria are better suited to capturing incomplete basolateral/lateral membranous staining patterns than existing algorithms for classifying breast cancer and have been adopted in various studies of gynecological malignancies [15,24,26]. Our findings support the practical value of this approach, as strong HER2 expression was predominantly found in the EC^hi^ group and was associated with worse survival, suggesting that careful HER2 scoring may help identify a biologically aggressive subgroup with potential therapeutic relevance [29].

In the DESTINY-PanTumor02 trial, the group with HER2 IHC 3+ showed the best ORR in endometrial cancer [8]. In addition, the group with HER2 IHC 3+ in endometrial cancer demonstrated significantly worse prognosis than the other groups in our study.

Bardia et al. described that, in the DESTINY-Breast06 trial, the therapeutic efficacy of T-DXd was maintained even in the groups with low or ultralow HER2 expression; however, that trial did not include patients with HER2 IHC 3+ [30]. Moreover, Chen et al. reported that resistance commonly emerges during treatment with T-DXd, particularly in the group with low HER2 expression, with such treatment frequently being associated with a marked decrease or complete loss of HER2 expression [31]. Taken together, these findings suggest that the HER2 IHC 3+ group is the most promising target population for T-DXd therapy, including from the perspective of resistance. In our study, HER2 IHC 3+ was also observed at a significantly higher frequency in the EC^hi^ group, indicating that, although both EC^hi^ and EC^lo^p53^ab^ groups exhibit p53-abnormal expression, they occupy distinct positions with respect to eligibility for T-DXd treatment.

Our results should also be interpreted in the context of the emerging HER2-low category in endometrial cancer. Recent studies have demonstrated that low HER2 expression and HER2 amplification are distributed across a broad spectrum of endometrial carcinomas and are not restricted to a single histological or molecular subtype [32,33]. This is clinically important because the efficacy of ADCs has expanded therapeutic interest beyond conventionally defined HER2-positive disease [34,35]. At the same time, these broader categories should not obscure the value of distinguishing HER2 IHC 3+ tumors. In our cohort, only HER2 IHC 3+, and not HER2-positive expression defined as IHC 2+/3+, was significantly associated with shorter PFS and OS, indicating that strong HER2 overexpression may identify the subgroup with the most aggressive cancer. Thus, while the use of the concept of HER2-low disease may enlarge the population considered for HER2-directed therapy, our findings suggest that the EC^hi^ group with HER2 IHC 3+ may represent the most compelling population for treatment stratification and prospective evaluation of HER2-targeted approaches [32,35,36].

Recently, Nakajima et al. analyzed a large, unselected cohort of endometrial carcinomas and demonstrated that HER2 positivity, including low-level expression, is relatively common across histological subtypes and that co-expression of HER2 and p53-abnormal identifies an aggressive subgroup with potential indications for ADC therapy [36]. In contrast, the present study specifically compared p53-abnormal, low-grade endometrial cancers with high-grade endometrial cancers and showed that strong HER2 IHC 3+ was markedly enriched in the EC^hi^ group but less frequent in the EC^lo^p53^ab^ group. This suggests that the EC^hi^ group should be prioritized when considering HER2-targeted strategies in those with p53-abnormal.

Recently, the cost-effectiveness of new treatments for cancer has become increasingly important. In this context, it is particularly valuable to develop prognostic markers for newly developed treatments.

Accordingly, the EC^hi^ group may represent a clinically meaningful subgroup in which HER2-targeted therapy, including T-DXd, could be particularly effective, although this requires further investigation. Despite the strengths of this study, this work involved various limitations, which should be mentioned here. First, this study had a small sample size, and further investigations with larger cohorts are warranted. In addition, this was a single-center, retrospective study, and HER2 assessment was performed on archival hysterectomy specimens, which may not fully capture HER2 heterogeneity in biopsy samples used in routine practice. Second, this retrospective cohort was accrued over a long inclusion period (1992–2022). During this time, pathological diagnostic criteria, tissue fixation and processing, staging systems, adjuvant treatment strategies, and follow-up practices likely changed at our institution. Because the study was not specifically designed or powered to evaluate temporal trends, we did not perform detailed analyses of HER2 score or survival by diagnosis era. These unmeasured time-dependent factors may therefore have influenced the observed clinicopathological associations and should be considered a major limitation of the present work. Third, TCGA-based molecular studies have established *POLE*-mutated endometrial carcinoma as a distinct molecular subgroup with an excellent prognosis. A systematic review and meta-analysis reported that approximately 8–9% of endometrial carcinomas harbor *POLE* mutations, the majority of which are endometrioid tumors and about half of which are FIGO grade 3 [37]. These data indicate that *POLE*-mutated tumors represent a small but clinically important subset that is particularly enriched among high-grade endometrioid carcinomas. In our cohort, none of the 13 EC^lo^ p53^ab^ tumors harbored pathogenic *POLE* hotspot mutations in exons 9 and 13. However, because *POLE* analysis was restricted to these hotspot regions because of technical and logistical constraints, rare non-hotspot variants outside these regions cannot be completely excluded; this should be considered a limitation of the present study. Meanwhile, in this study, HER2 IHC 3+ was significantly associated with older age (≥60 years) and LVSI. However, in endometrial carcinoma, HER2 overexpression has been reported more frequently in high-grade tumors and in association with other high-risk histopathological features, including deep myometrial invasion and LVSI [23,38], whereas a consistent age-related increase in HER2 expression has not been clearly demonstrated to date. Taken together, these observations indicate that HER2 status, patient age, and LVSI are all relevant clinicopathological factors in risk assessment; further studies are warranted to clarify their independent and combined impacts on prognosis and on the selection of candidates for HER2-targeted therapies.

## 5. Conclusions

In this study, we investigated the difference in HER2 IHC between the EC^lo^p53^ab^ group and the EC^hi^ group. Our results showed that HER2 IHC 3+ was significantly more frequent in the EC^hi^ group than in the EC^lo^p53^ab^ group. In addition, HER2 IHC 3+ was associated with shorter PFS and OS. These findings suggest that the EC^hi^ group represents a biologically and clinically distinct subset from the EC^lo^p53^ab^ group, in which HER2-targeted therapy, including T-DXd, may be particularly promising. Given the emerging importance of cost-effective, biomarker-driven strategies, HER2 IHC 3+ could serve as a useful marker to identify candidates for such therapies. However, our study is limited by its small sample size, and larger prospective studies are required to validate these observations and to clarify the optimal integration of HER2-targeted therapy into the treatment algorithm for endometrial cancer.

## Figures and Tables

**Table 1:** Clinicopathological characteristics of the 70 endometrial cancer patients.

	Low-Grade Endometrial Cancer with Wild-Type p53 Expression (*n* = 32)	Low-Grade Endometrial Cancer with p53-Abnormal Expression (*n* = 13)	High-Grade Endometrial Cancer (*n* = 25)
Age, *n* (%)			
<60 years	13 (40.6%)	4 (30.8%)	2 (8.0%)
≥60 years	19 (59.4%)	9 (69.2%)	23 (92.0%)
Myometrial invasion, *n* (%)			
<1/2	12 (37.5%)	9 (69.2%)	12 (48.0%)
≥1/2	20 (62.5%)	4 (30.8%)	13 (52.0%)
FIGO 2023 stage, *n* (%)			
early (I + II)	7 (21.9%)	10 (76.9%)	10 (40.0%)
advanced (III + IV)	25 (78.1%)	3 (23.1%)	15 (60.0%)
Distant metastasis, *n* (%)			
present	4 (12.5%)	1 (7.7%)	8 (32.0%)
absent	28 (87.5%)	12 (92.3%)	17 (68.0%)
Lymph node metastasis, *n* (%)			
present (*n* = 15)			
ITCs	1 (3.4%)	0 (0%)	0 (0%)
micrometastasis	2 (6.9%)	1 (7.7%)	4 (19.0%)
macrometastasis	1 (3.4%)	1 (7.7%)	5 (23.8%)
absent (*n* = 48)	25 (86.3%)	11 (84.6%)	12 (57.2%)
Uterine cervical invasion, *n* (%)			
present	4 (12.5%)	2 (15.4%)	2 (8.0%)
absent	28 (87.5%)	11 (84.6%)	23 (92.0%)
Peritoneal cytology, *n* (%)			
positive	5 (15.6%)	1 (7.7%)	11 (44.0%)
negative	27 (84.4%)	12 (92.3%)	14 (56.0%)
Lymphovascular invasion, *n* (%)			
none	16 (50.0%)	10 (76.9%)	12 (48.0%)
focal	8 (25.0%)	2 (15.4%)	7 (28.0%)
substantial	8 (25.0%)	1 (7.7%)	6 (24.0%)
Hysterectomy, *n* (%)			
yes	32 (100%)	13 (100%)	25 (100%)
no	0	0 (0%)	0 (0%)
Lymphadenectomy, *n* (%)			
yes	29 (90.6%)	13 (100%)	21 (84.0%)
no	3 (9.4%)	0 (0%)	4 (16.0%)
Recurrence, *n* (%)			
yes (*n* = 17)	2 (6.3%)	3 (23.1%)	12 (54.5%)
no (50)	30 (93.7%)	10 (76.9%)	10 (45.5%)

FIGO: International Federation of Gynecology and Obstetrics; ITC: isolated tumor cell.

**Table 2:** Distribution of HER2 immunohistochemical expression among the endometrial cancer groups.

	Low-Grade Endometrial Cancer with Wild-Type p53 Expression (*n* = 32)	Low-Grade Endometrial Cancer with p53-Abnormal Expression (*n* = 13)	High-Grade Endometrial Cancer (*n* = 25)
HER2 immunohistochemical expression			
3+	6 (18.8%)	4 (30.8%)	18 (72.0%)
2+	14 (43.8%)	8 (61.5%)	6 (24.0%)
1+	12 (37.4%)	1 (7.7%)	1 (4.0%)
0	0 (0%)	0 (0%)	0 (0%)

HER2: Human Epidermal Growth Factor Receptor 2.

**Table 3:** HER2 immunohistochemical expression (3+) among the groups.

	Low-Grade Endometrial Cancer with Wild-Type p53 Expression (*n* = 32)	Low-Grade Endometrial Cancer with p53-Abnormal Expression (*n* = 13)	High-Grade Endometrial Cancer (*n* = 25)
HER2 immunohistochemical expression			
3+	6 (18.8%)	4 (30.8%) *	18 (72.0%) *
0–2+	26 (81.2%)	9 (69.2%) *	7 (28.0%) *

* *p* < 0.05, low-grade endometrial cancer with p53-abnormal expression versus high-grade endometrial cancer. HER2: Human Epidermal Growth Factor Receptor 2.

**Table 4:** HER2-positive immunohistochemical expression (2+ and 3+) among the groups.

	Low-Grade Endometrial Cancer with Wild-Type p53 Expression (*n* = 32)	Low-Grade Endometrial Cancer with p53-Abnormal Expression (*n* = 13)	High-Grade Endometrial Cancer (*n* = 25)
HER2 immunohistochemical expression			
positive (2+ and 3+)	20 (62.5%)	12 (92.3%)	24 (96.0%)
negative (0 and 1+)	12 (37.5%)	1 (7.7%)	1 (4.0%)

HER2: Human Epidermal Growth Factor Receptor 2.

## Data Availability

The datasets used and/or analyzed during the current study are available from the corresponding author on reasonable request.

## Supplementary Materials

The following supporting information can be downloaded at: https://www.mdpi.com/article/10.3390/cancers18162638/s1. Figure S1: Representative immunohistochemical staining for PMS2 and MSH6 in low-grade endometrial cancer with p53-abnormal expression (EC^lo^p53^ab^). (A) PMS2 shows retained nuclear staining in tumor cells and surrounding normal cells. (B) MSH6 shows retained nuclear staining in tumor cells and surrounding normal cells. These patterns illustrate mismatch repair proficiency in the EC^lo^p53^ab^ group.; Figure S2: Distribution of HER2 immunohistochemical scores by tumor group. Bars show the percentages of cases with HER2 IHC 1+, 2+, and 3+ in low-grade endometrial cancer with p53 wild-type expression (EC^lo^p53^wt^), low-grade endometrial cancer with p53-abnormal expression (EC^lo^p53^ab^), and high-grade endometrial cancer (EC^hi^). The figure highlights the higher proportion of HER2 IHC 3+ tumors in the EC^hi^ group compared with the EC^lo^p53^wt^ and EC^lo^p53^ab^ groups; Table S1: Associations of HER2 immunohistochemical expression (3+) with older age and substantial lymphovascular space invasion

## Abbreviations

IHC	immunohistochemical expression
HER2	Human Epidermal Growth Factor Receptor 2
EC^lo^p53^wt^	low-grade endometrial cancer with wild-type p53 expression
EC^lo^p53^ab^	low-grade endometrial cancer with p53-abnormal expression
EC^hi^	high-grade endometrial cancer
TCGA	The Cancer Genome Atlas
FIGO	International Federation of Gynecology and Obstetrics
ADC	Antibody–drug conjugate
T-DXd	trastuzumab deruxtecan
ORR	overall response rate
HE	hematoxylin and eosin
LVSI	lymphovascular space invasion
PFS	progression-free survival
OS	overall survival
ITC	isolated tumor cell
EGFR	Epidermal Growth Factor Receptor
