# Supplementary material for: Clinicopathological Differences in HER2 Immunohistochemical Expression Between Low-Grade Endometrial Cancer with p53-Abnormal Expression and High-Grade Endometrial Cancer
Source: Cancers (Basel). 2026 Aug 15;18(16):2638. doi: 10.3390/cancers18162638 (PMC13511264; doi:10.3390/cancers18162638)
Supplement: Supplementary file 1 [file cancers-18-02638-s001.zip › cancers-4443934-supplementary.pdf]

cancers

Supplementary Materials

Clinicopathological Differences in HER2 Immunohistochemical Expression Between Low-Grade Endometrial Cancer with p53-Abnormal Expression and High-Grade Endometrial Cancer

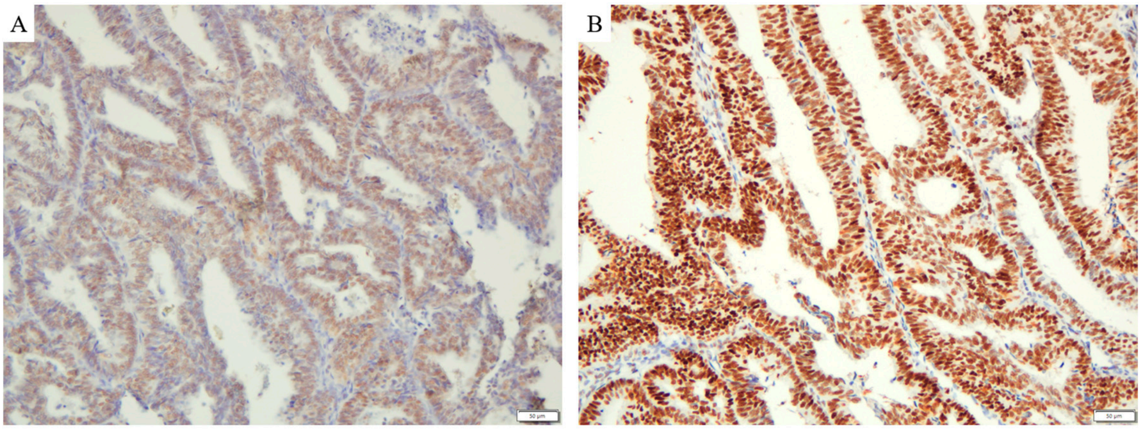

Figure S1. Representative immunohistochemical staining for PMS2 and MSH6 in low-grade endometrial cancer with p53-abnormal expression ($EC^{lo}p53^{ab}$). (A) PMS2 shows retained nuclear staining in tumor cells and surrounding normal cells. (B) MSH6 shows retained nuclear staining in tumor cells and surrounding normal cells. These patterns illustrate mismatch repair proficiency in the $EC^{lo}p53^{ab}$ group.

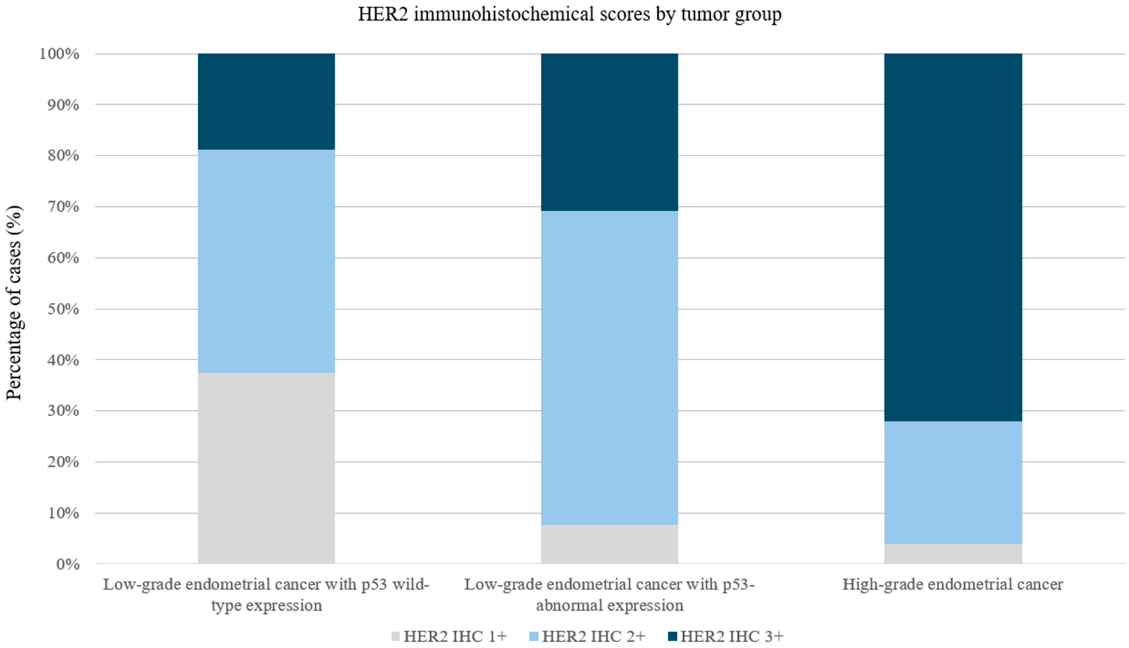

Figure S2. Distribution of HER2 immunohistochemical scores by tumor group. Bars show the percentages of cases with HER2 IHC 1+, 2+, and 3+ in low-grade endometrial cancer with p53 wild-type expression ($EC^{lo}p53^{wt}$), low-grade endometrial cancer with p53-abnormal expression ($EC^{lo}p53^{ab}$), and high-grade endometrial cancer (EC^{hi}). The figure highlights the higher proportion of HER2 IHC 3+ tumors in the EC^{hi} group compared with the $EC^{lo}p53^{wt}$ and $EC^{lo}p53^{ab}$ groups.

 https://doi.org/10.3390/cancers18162638

Table S1. Associations of HER2 immunohistochemical expression (3+) with older age and substantial lymphovascular space invasion.

	Age < 60, n (%)	Age ≥ 60, n (%)	p-value	LVSI none and focal, n (%)	LVSI substantial, n (%)	p-value
HER2 immunohistochemical expression						
3+	1 (5.3%)	27 (52.9%)		18 (32.7%)	10 (66.7%)	
0-2+	18 (94.7%)	24 (47.1%)	0.0003	37 (67.3%)	5 (33.3%)	0.0174

HER2: Human Epidermal Growth Factor Receptor 2, LVSI: lymphovascular space invasion

Disclaimer/Publisher's Note: The statements, opinions and data contained in all publications are solely those of the individual author(s) and contributor(s) and not of MDPI and/or the editor(s). MDPI and/or the editor(s) disclaim responsibility for any injury to people or property resulting from any ideas, methods, instructions or products referred to in the content.

https://doi.org/10.3390/cancers18162638
